# Computational Insights into the Allosteric Modulation of a Phthalate-Degrading Hydrolase by Distal Mutations

**DOI:** 10.3390/biom13030443

**Published:** 2023-02-26

**Authors:** Ran Xu, Yiqiong Bao, Mengrong Li, Yan Zhang, Lili Xi, Jingjing Guo

**Affiliations:** 1 College of Life Sciences, Nanjing Agricultural University, Nanjing 210095, China; 2 School of Pharmacy, Guizhou University of Traditional Chinese Medicine, Guiyang 550025, China; 3 Office of Institution of Drug Clinical Trial, The First Hospital of Lanzhou University, Lanzhou 730020, China; 4 Centre in Artificial Intelligence Driven Drug Discovery, Faculty of Applied Science, Macao Polytechnic University, Macao 999078, China; 5 Engineering Research Centre of Applied Technology on Machine Translation and Artificial Intelligence, Macao Polytechnic University, Macao 999078, China

**Keywords:** esterases, phthalate esters, rational design of proteins, distal mutations, molecular dynamics simulation, toxicity

## Abstract

Phthalate esters (PAEs) are a ubiquitous kind of environmental endocrine that disrupt chemicals, causing environmental and health issues. EstJ6 is an effective phthalate-degrading hydrolase, and its mutant with a combination of three non-conservative distal mutations has an improved activity against PAEs with unknown molecular mechanisms. Herein, we attempt to fill the significant gap between distal mutations and the activity of this enzyme using computational approaches. We found that mutations resulted in a redistribution of the enzyme’s preexisting conformational states and dynamic changes of key functional regions, especially the lid over the active site. The outward motion of the lid upon the mutations made it easier for substrates or products to enter or exit. Additionally, a stronger substrate binding affinity and conformational rearrangements of catalytic reaction-associated residues in the mutant, accompanied by the strengthened communication within the protein, could synergistically contribute to the elevated catalytic efficiency. Finally, an attempt was made to improve the thermostability of EstJ6 upon introducing a distal disulfide bond between residues A23 and A29, and the simulation results were as expected. Together, our work explored the allosteric effects caused by distal mutations, which could provide insights into the rational design of esterases for industrial applications in the future.

## 1. Introduction

**Figure 1:**
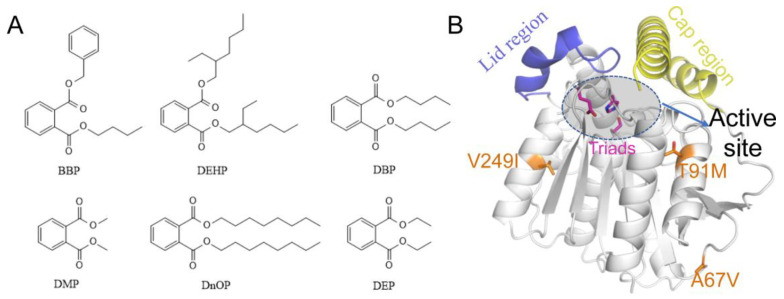
The 2D structures of six common PAEs and the 3D structure of EstJ6. (**A**) The 2D structures of six common PAEs including BBP, DEHP, DBP, DMP, DnOP, and DEP. (**B**) Overview of EstJ6 stereo structure and mutation sites for ET2.2. Magenta sticks represent triads S146, E240, and H270. Essential motifs including lid and cap regions are colored purple and yellow, respectively. The substrate binding pocket is highlighted by an oval.

Phthalate esters (PAEs) are extensively used in plastic manufacturing to increase the flexibility and durability of materials [1,2,3]. Usually, PAEs are combined with materials physically so that they are easily released into the air, water, and soil during processes of application, transportation, and stockpile [4,5] and have become a major environmental hazard. They can stably exist and accumulate in the environment because of their structural stability [6]. PAEs in the environment derive mostly from artificial synthesis, and a very small part is from plant synthesis; for example, freshwater algae and cyanobacteria are capable of producing dibutyl phthalate (DBP) [7,8]. Six kinds of PAEs (Figure 1A), including dibutyl phthalate (DBP), diethyl phthalate (DEP), diethyl hexyl phthalate (DEHP), butyl benzyl phthalate (BBP), dioctyl phthalate phthalate (DnOP), and dimethyl phthalate (DMP), are listed as priority pollutants by the US Environmental Protection Agency [9]. DEHP with large sidechains and DBP with short sidechains are two of the most widely used PAEs and are more contaminated than other types [10]. Generally, the exposure routes of humans to PAEs include intrusion into the digestive system via the food chain, inhaling into the lungs by breathing, and direct skin contact [9,11]. Afterward, they quickly participate in the metabolism of the body and pose a serious threat to human health [10]. At very low concentrations, they interfere with the endocrine systems of humans and animals, even causing male reproductive abnormalities and precocious puberty in children. Each year, the global use of PAEs is about eight million tons, and this number still continues to grow [11]. Therefore, the development of an efficient, economical, and environmentally friendly approach to decontaminate the PAE-polluted environment is needed [12].

Under the no-interference condition, the degradation process of PAEs, via hydrolysis or photolysis is time-consuming. Biodegradation is the principal approach to removing PAEs in the environment, with the advantages of high efficiency, low consumption, and environmental safety. Especially the leading role of bacterial degradation in the removal of phthalates has been widely explored [13,14]. A total of 99% of natural bacteria are unable to be cultured under experimental conditions; however, meta-genome technology resolves this obstacle, to a large extent, in excavating the esterase resource [15,16]. In terms of the general metabolic pathway of phthalates, it can be divided into two main steps, namely, the hydrolysis of the two ester bonds [17]. Some strains have the ability to hydrolyze phthalate esters of the phthalic acid monoester but cannot further utilize the latter. Based on the above problems, researchers chose the sludge of the lotus pond as the object with which to extract the total DNA in order to establish a meta-genome library and unearth some new kinds of esterases [18]. Interestingly, one novel esterase named EstJ6 was discovered, which possessed the ability to hydrolyze both ester bonds of PAEs, sequentially.

Generally, the native esterases have a limited hydrolysis ability and thermostability, which cannot satisfy industry development demands. For the purpose of acquiring more practical esterases, directed evolution emerges as time requires. An error-prone PCR was performed to construct a library of EstJ6 mutants, followed by substrate screening to obtain the optimal mutants. Finally, ET2.2 with mutations (A67V, T91M, and V249I) was obtained with the strongest stability and the best function [19]. Due to the absence of the EstJ6 crystal structure, homology modeling assisted us in constructing one credible substitute (Figure 1B). Based on the structure of EstJ6, the entrance of the active site could be roughly divided into the lid and cap regions. The difference is that the former was relatively static while the latter was very flexible. According to the modeled structure of EstJ6, the mutated residues in ET2.2 are away from the active pocket. However, it is not clear how these distal mutations increase enzyme activity.

At present, research on the biodegradation of PAEs mainly stays on the screening of degrading strains, but few efforts have focused on the implied molecular mechanisms of related PAE degrading enzymes. Classical biological methods have many limitations in the study of protein functions, and the most nonnegligible point is that they cannot accurately describe the kinetic behavior of proteins. Now, the development of computational biology technology provides us with a new perspective with which to understand protein functions from both conformational and dynamic changes upon mutation [20].

In many cases, remote mutations could change the non-covalent interaction network within proteins, which could result in a redistribution of the enzyme’s preexisting conformational states or changes in the conformational dynamics of key functional structural elements, such as loops and covers around the active sites [21], and further affects the function of enzymes. In this study, the allosteric regulation of EstJ6 upon three combined distal mutations was uncovered through the use of computational methods, such as homology modeling, molecular docking, and molecular dynamics simulations. DBP, the best substrate of EstJ6 and ET2.2, was applied here as a probe to analyze the relationship between structure and the function of the phthalate-degrading hydrolase EstJ6. Our results not only provide insights into the rational design of PAE-degrading hydrolases based on EstJ6 but might also contribute to the expansion of existing enzymes upon distal mutations in the future.

## 2. Materials and Methods

### 2.1. Sequence Alignment and Co-Evolution Analysis

The sequence of EstJ6 was collected from the online site of the National Center for Biotechnology Information (NCBI). Based on the sequence similarity, the first nine sequences (sequence similarity to EstJ6 ≥ 57%) were collected from uniport [22] (https://www.uniprot.org/, accessed on 19 March 2021) through the use of the Blast module. The Clustalw server (https://www.genome.jp/tools-bin/clustalw, accessed on 24 May 2021) was then applied for further sequence alignment, and then ESPript [23] was used for figure prettification. By means of evolutionary sequence covariation, the EV-couplings website (https://evcouplings.org/, accessed on 19 March 2021) was employed to predict co-evolution pairs, conservative residues, and the mutational effects of EstJ6 with default parameters. The sequence alignment result obtained from the Ev-coupling website was uploaded to WebLogo3 (https://weblogo.threeplusone.com/, accessed on 16 November 2022) to generate a sequence logo figure.

### 2.2. Homology Modeling and Molecular Docking

The initial three-dimensional structure of EstJ6 (residues 2-297) was constructed using the automated Swiss-Model web service. A template (PDB ID: 6Y9K [24]) with the highest sequence identity (86.53%) was used. The rationality of the structure was verified by the Ramachandran plot. In order to obtain the mutant ET2.2, we performed in silico mutations using PyMOL [25]. The molecular docking of EstJ6 and ET2.2 with DBP was then carried out by AutoDock Vina [26]. The semi-flexible docking method was used, which meant that the protein was rigid and the ligand flexible [27]. The optimal binding modes were searched within a box of 20 × 20 × 20 Å^3^ along with the triads and oxyanion-hole residues as the box center. The best binding poses for EstJ6 and ET2.2 with DBP were preserved and presented.

### 2.3. Conventional Molecular Dynamics Simulation

All-atom classic molecular dynamics simulations (CMD) for EstJ6, ET2.2, EstJ6-DBP, and ET2.2-DBP (hereafter referred to as WT-apo, Mut-apo, WT-com, and Mut-com) were performed using the AMBER 18 suite [28] for three 500-ns replicates in explicit water. Tleap [28] was used to generate topology and coordinate files using the ff14SB force field [29]. Na^+^ or Cl^−^ ions were added to maintain the neutrality of the systems. The models were solvated in a rectangular box filled with TIP3P water molecules [30]. The distance between the solute and the box borders was at least 10 Å. The files for molecular dynamics simulation were ready. Subsequently, each system was energetically minimized through a multi-step process. Then, these systems were gradually heated from 0 to 300 K by 10,000 steps, followed by a 200-ps equilibration which was performed at 300 K and 1.0 atm for all systems. Afterward, MD production simulations were performed. The time step for all production simulations was set to 2 fs, and a nonbonded cutoff of 8 Å was used with periodic boundary conditions. The particle mesh Ewald (PME) method [31] was used to handle the long-range electrostatic interactions. All bonds involving hydrogen atoms were constrained using the SHAKE algorithm [32]. The coordinates were saved every 4 ps.

Considering the large pocket size of EstJ6, it is difficult to capture a stabilized transition state of DBP and the residues that were involved in the catalytic reaction at the active site. Hence, DBP was confined to a certain space by conducting simulations with distance restraints. In addition, the states of triads were also considered. In detail, three distance restraints were applied with a 5.5-Å upper bound during our restrained simulations in order to simulate the catalytic state: (i) DBP and S146; (ii) S146 and H270; (iii) H270 and E240. Specific atoms in restrictions included a certain carbonyl carbon of DBP and atoms in triads, which were involved in the hydrogen-bond network. The harmonic biased potential with a force constant of 32 kcal/mol/Å^2^ was used. The WT-com and Mut-com systems were simulated for three 600-ns replicates.

### 2.4. Trajectory Analyses

The root-mean-square deviations (RMSDs) of all Cα atoms were calculated during the simulation time and referred to the initial structure to evaluate the structural stability and the equilibrium state of MD simulations. The residual flexibilities of these proteins were detected by calculating Cα root-mean-square fluctuations (RMSFs). The last 100-ns simulations used the average structures as references. Statistical analyses, such as the residue–residue correlation map, *R*_g_, cluster analysis, and principal component analysis (PCA), were performed over the last 100-ns trajectories using the CPPTRAJ module [33] of the AMBER 18 software. The residues within 5 Å of DBP were considered pocket residues. The binding free energy between the protein and ligand was calculated using the MM-GBSA approach [34]. Dynamical network analysis [35] was carried out using VMD [36] with the default settings.

### 2.5. Disulfide-Bond Design

Disulfide by Design2 (DbD2) [37] (http://cptweb.cpt.wayne.edu/DbD2/index.php, accessed on 29 December 2021) is an online version of the original DbD website for assessing disulfide bonds. The homology-modeling structure of Estj6 was uploaded to DbD2 as the template. The criterion for the χ3 torsion angles was specified to the smallest range of −87°/+97° ± 30° and for the C_α_-C_β_-C_γ_ bond angles of 114.6 ± 10°. Among all candidates, we chose the more promising residue pairs for disulfide bond design based on the sequence and structure analysis.

## 3. Results

### 3.1. Sequential and Structural Analyses of EstJ6

**Figure 2:**
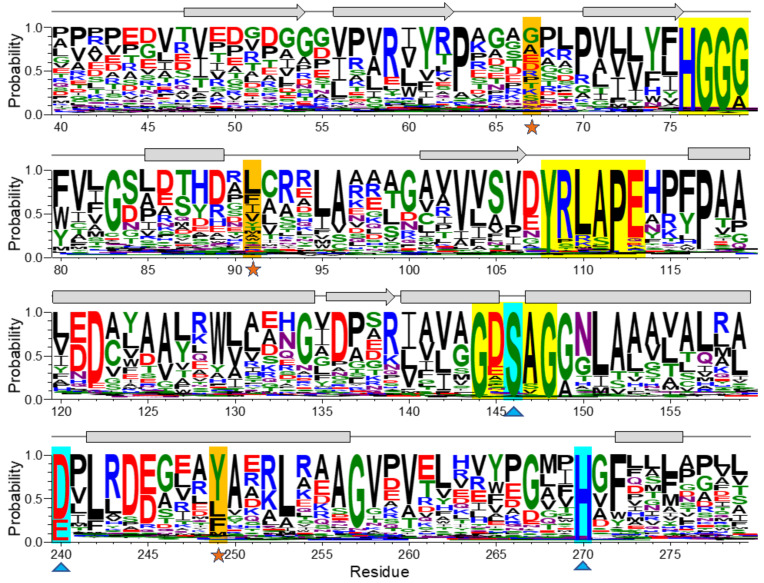
Graphical representation of sequence conservation of partial EstJ6. The sequence logo was generated by the WebLogo3 server after sequence alignment. The mutated sites, triads, and three conservative motifs are highlighted in orange, cyan, and yellow, respectively. The size of the logos represents the relative frequency of each residue.

Both sequence alignment and co-evolution analysis were performed to recognize highly conservative residues along with the residue–residue coupling information of EstJ6. The alignment result involving high similarity sequences shown in Figure S1 unveils some crucial conservative residues in EstJ6, such as the oxyanion hole residues 75-HGGG-80, conservative helical region 143-GXSXG-149 [38], and catalytic triads (S146, E240, and H270) [18], which play essential roles in ligand binding and catalytic process for family IV of hydrolyses. We then employed WebLogo 3 [39] to perform a sequence logo generation based on a multiple sequence comparison of EstJ6, which was obtained from Ev-coupling to investigate the residue conservativeness, and the results are shown in Figure 2 and Figure S2. Not surprisingly, residues of the mentioned two crucial motifs and catalytic triads had a high frequency (Figure 2 and Figure S2 and Table S1). For example, the first X in the GXSXG conserved motif was customarily composed of acid residues, such as glutamate (11.25%) and aspartate (62.27%). As for the second X, its frequency of alanine was 82.75%. The occurrence probability of residue 240 for acidic residues in the catalytic triads, including glutamate and aspartate, was 23.88% and 74.13%, respectively. During the hydrolysis process, the position determination of H270 was assisted by E240 via a hydrogen-bond interaction [18,40]. Noteworthy, E240 and H270 belong to coupling evolutionary residue pairs according to the Ev-coupling analysis. As for the three mutational sites, all of them had a low occurrence frequency during the evolution process (Figure 2 and Table S1).

To further investigate the structural characteristic of EstJ6, its three-dimensional structure was modeled using the homology modeling method [41] within the structure (PDB ID: 6Y9K [24]; sequence identity to EstJ6: 86.53%) as a template (Figure 1B). Ramachandran plot (Figure S3) displays 90.9% of the residues that reside in the most favored regions, which indicates the conformational rationality of the modeling product. EstJ6 presents a typical α/β hydrolase folding structure and possesses typical cap and lid regions, which are vital for substrate selectivity and binding [42]. The binding pocket is mainly formed by hydrophobic residues (Figure S4), which is beneficial for the binding of PAEs, which are hydrophobic molecules.

Referring to the structure of EstJ6, three mutations of ET2.2 (A67V, T91M, and V249I) are all distal from the active site. Mutational analysis from an online server named Ev-coupling predicted the effective strength of these three mutations: T91M (3.942), A67V (−2.782), and V249I (−1.384) (<0, damaging substitution; =0, neutral substitution; >0, beneficial substitution). Although two of them might be negative in the function of EstJ6 in the condition of a single mutation, wet experiments suggest that their combination is beneficial to the esterase. For the sake of exploring the local structure influence caused by mutations, we compared the property of these mutation sites. After mutation, all three sites reinforced their hydrophobicity, and hence we speculated that these changes might affect the overall conformational dynamics of EstJ6 and further its function. To confirm our speculation, molecular dynamic simulations were performed to gain atomic-level insights into the influence of the combination of three distal mutations on the structure and dynamics of EstJ6.

### 3.2. Global and Local Conformational Variations of EstJ6 Induced by Mutations and Substrate Binding

To explore the allosteric regulation mechanism of distal mutations on EstJ6, four total systems were considered for molecular dynamic (MD) simulations, including wild type and mutated EstJ6 with and without DBP (name as WT-apo, WT-com, Mut-apo, and Mut-com). Each system was conducted for three 500-ns simulation replications. The MD simulation allows the protein to be completely relaxed in the solvent, collecting dynamic information that is unattainable from a static situation [43]. First, all MD trajectories were monitored by the root-mean-square deviations of Cα atoms (Cα RMSDs) referring to the starting structure to evaluate whether the systems were stable and converged (Figure S5). Almost all the simulations were equilibrated after 300-ns, undulating around 1.5 Å~2 Å, which indicated that the structures did not deviate much from the original structure and were stable during the simulations.

**Figure 3:**
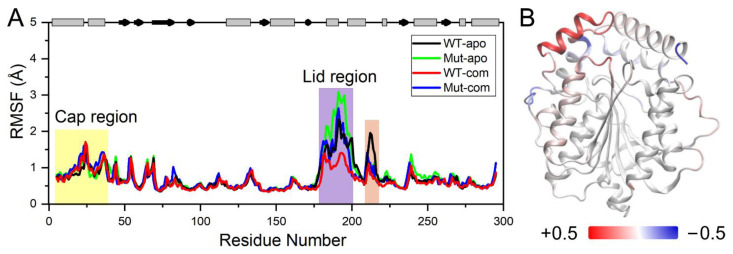
System stability of all systems. (**A**) Cα RMSFs based on the last 100-ns MD trajectories for each system used their average conformations as references. The results of all systems are averaged over the three replicate simulations. (**B**) Changes in RMSF upon mutations displayed on the apo structure according to a color scale (lower and higher flexibilities depicted in blue and red, respectively).

In order to further investigate the flexibility of individual residues, the analysis of root-mean-square fluctuations of Cα atoms (Cα RMSFs) for the last 100-ns simulations was performed (Figure 3A). The lid (residues 182–199) over the active site exhibits the highest RMSF values in all the systems. Our results suggest that ligand binding makes the lid more rigid, while mutations result in a more flexible lid region. The subsequent peak in WT-apo involved residues G211-S216 and was reduced in other systems, which might be advantageous to maintain the stability of the hydrophobic pocket. In order to intuitively observe how the changes caused by mutations in RMSF are distributed spatially, we mapped the RMSF differences between the two apo systems onto the protein structure (Figure 3B). A significant increase in the flexibility of the lid region could be easily observed. In addition, the first helix of the cap region and a loop close to the entrance of the ligand binding pocket also became slightly more flexible in Mut-apo, while regions deeper in the pocket were found with moderately enhanced rigidification.

**Figure 4:**
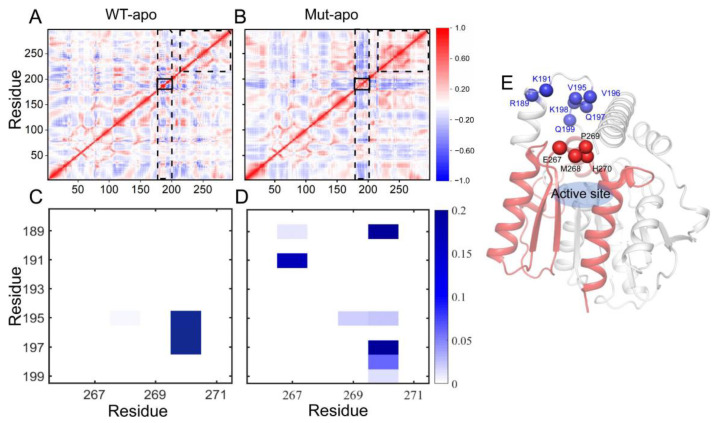
The residue–residue correlation information of the entire protein and the contact information of the pocket entrance regions in the two apo systems. (**A**,**B**) Dynamical cross-correlation maps for the Cα atom pairs according to the last 100-ns MD trajectories: WT-apo (**A**) and Mut-apo (**B**). Areas with strong variations between apo systems are emphasized with black boxes. (**C**,**D**) The contact map between the lid region and residues 265–272: WT-apo (**C**) and Mut-apo (**D**). (**E**) The overall structure of EstJ6. The pocket entrance residues are indicated by blue and red spheres, and residues 200–297 are depicted in red.

Proteins are dynamic, and the relationships among residues play important roles in protein functions [44], as well as the allosteric regulation of enzymes [45,46]. Through constructing a dynamic cross-correlation matrix (DCCM) [47], the correlations of each pair of Cα atoms were intuitively vivid. The results of DCCMs for EstJ6-apo and ET2.2-apo were calculated based on the last 100-ns trajectories and are presented in Figure 4A,B. The correlation map of WT-apo is quite disordered, while the color deepens in most areas after mutations, revealing the increased residue–residue communication within the protein upon mutations. In detail, residues become more positively correlated with neighboring ones, indicating the enhanced self-correlation of some regions, especially residues 220–297 containing triad residues E240 and H270. Moreover, an obvious negative correlation between the areas containing residues 175–200 and most of the rest was observed, which might be related to the enhanced stretching movements of the lid region. Additionally, the internal correlations among the lid residues were enhanced positively, which might help to control the pocket in a more coordinated way.

To further delve into the patency for substrate entry into the binding pocket, we analyzed the contact information between the lid region and a motif involving residues from 265 to 272. As presented in Figure 4C,D, for the WT-apo system, there are very high contact intensities between H270 with residues 195–197 of the lid region. These contacts can generate a certain spatial barrier for DBP entering into the pocket, whereas most of these contacts disappeared or were weakened upon mutations and were replaced by several low-frequency contacts. Additional contacts, such as 191–267, 189–267, and 189–270, might be formed when the lid region stretches up away from the pocket.

### 3.3. Mutations Increase the Flexibility of the Lid Region Resulting in a More Open Pocket

**Figure 5:**
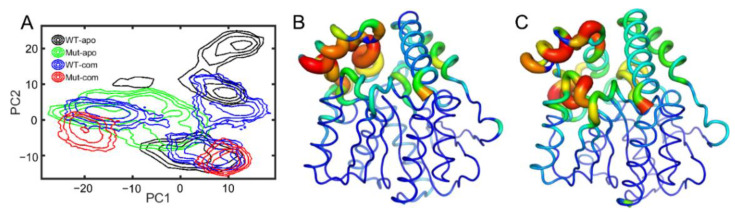
Free energy profiles over the first two principal components (PC1 and PC2) in all systems: WT-apo, Mut-apo, WT-com, and Mut-com (**A**). The displacements of the protein along PC1 (**B**) and PC2 (**C**) are mapped onto the structure. The detailed motions along the two PCs are displayed in Supplementary Videos S1 (PC1) and S2 (PC2). In the videos, the lid and cap regions are colored purple and yellow, respectively. Additionally, the Cα atoms of essential pocket residues are depicted as green spheres, including oxygen hole residues and triads.

Next, to depict the conformational discrepancies of EstJ6 upon DBP binding and mutations, a principal component analysis (PCA) was performed for the last 100-ns MD trajectories of all the systems considering three replicates together. The results are displayed in the free-energy surface using the first two principal components (called PC1 and PC2). As illustrated in Figure 5A, all systems exhibited decentralized or broad conformational sampling, indicating that systems from three independent replicates were not trapped in a particularly long-lived metastable state. The structure of the WT-apo was mainly distributed along PC1, while the others were along PC2. To intuitively recognize the global motions captured by PC1 and PC2, the residue displacements were mapped onto the protein structure (Figure 5B,C), and the conformational motions along PC1 and PC2 are also shown in Supplementary Videos S1 and S2.

The most mobile region to be captured by both PC1 and PC2 was the lid region (Figure 5B,C), which is consistent with RMSF analysis (Figure 3A). In detail, according to Supplementary Video S1, PC1 mainly captures the open-to-closed motion of the lid. The inward or closure motion of the lid was accompanied by negligible changes in the rest of the protein. The lid region, which controls the opening and closing of the pocket, plays an important role in substrate recognition and binding. The open state should be easier for the entrance of substrates and the release of the product, while a relatively stable and closed active site might benefit substrate binding and further catalytic reaction. Additionally, PC2 illustrates the stretch and shrink motion of the lid region, as well as the overall conformational rearrangement of EstJ6, as displayed in Supplementary Video S2. Finally, a more confined pocket could be noticed along PC2. As a result, WT-apo prefers a smaller binding pocket, which might not be conducive to ligand binding or positioning.

Based on the PCA results, the global motions of EstJ6 include the opening of the entrance and the expansion of the inside space of the pocket. For apo EstJ6, mutations resulted in the opening of the pocket with an intermediate-size space. Upon substrate binding, the lid swung between open and closed states, and the closed one preferred the larger inside space of the active site, especially for the Mut-com one. Therefore, the opening state upon mutations is beneficial for the entering of ligands; meanwhile, the ligand binding requires a spacious interior space.

**Figure 6:**
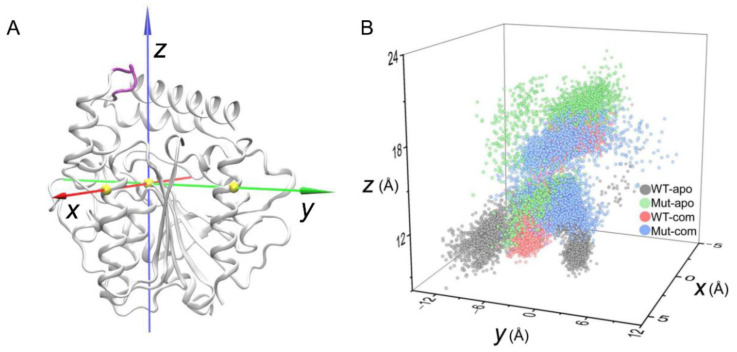
Displacements of the lid region according to the self-defined coordinate system. (**A**) The self-defined coordinate system: the Cα atom of S146 is set as the origin, and the x- and y-axes process through the Cα atoms of Q244 and D284, respectively. The three Cα atoms are shown as yellow spheres. The lid region (Cα atoms of residues 192–196, colored in purple) are prepared for vector calculations. (**B**) The plot of the position of the lid region according to the defined coordinate system.

As discussed above, the high flexibility of the lid region was unveiled. To further investigate the opening and closing of the active-site entrance, the displacements of the lid were monitored according to a self-defined coordinate system (Figure 6B). The open-closed motion is mainly captured by the *z*-axis, while the *x/y*-axis captures the twist motion. During simulations, the distinct distribution of the lid conformations in the four systems could be observed. As indicated in Figure 6, the lid region in the Mut-apo system presents more decentralized conformational sampling compared to those in other systems. The entrance in WT-apo was closed most before mutations resulted in an outward movement along the *z*-axis, while ligand binding induced the lid to have a larger motion along the positive direction of the *y*-axis, especially the Mut-com system. It seems that the lid region of Mut-com tended to cover above the pocket. We can conclude that mutations and ligand binding do influence the lid motion to regulate the pocket opening and closing, which is consistent with the PCA result.

### 3.4. Effects of Distal Mutations on the Active Site

**Figure 7:**
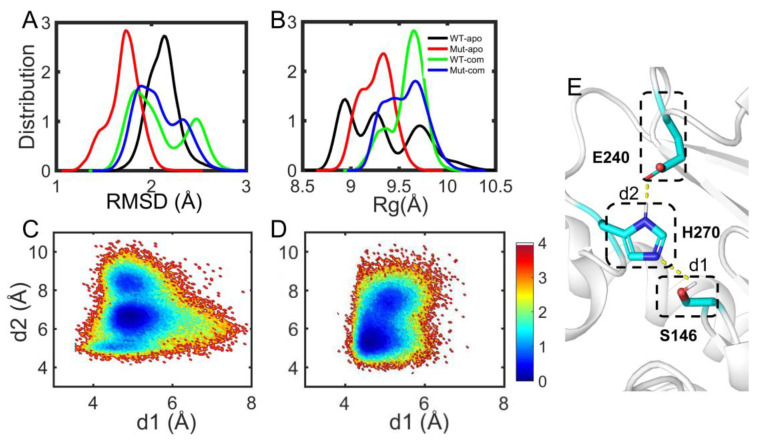
Substrate binding pocket analyses. The distribution of RMSD (**A**) and *R*_g_ (**B**) of hydrophobic residues in the binding site. The free energy landscape distribution of the side-chain distances of the catalytic triad including S146-H270 (d1) and E240-H270 (d2) with lower energy minima basins colored in deep blue for WT-com (**C**) and Mut-com (**D**). (**E**) The local structure and hydrogen-bond network of the active site involving the catalytic triads.

As discussed above, the overall and partial structural stabilities and motions of the EstJ6 system behave differently upon ligand binding and/or mutations, especially the open-closed motion of the lid over the active site. To further explore the properties of the active pocket, the stability of hydrophobic residues within 5 Å of DBP was analyzed (Figure 7A,B and Table S2). As can be seen, mutations result in a more compact and stable substrate binding pocket for apo EstJ6. Due to the low polarity of DBP and a relatively large pocket of EstJ6, the residues of the pocket are disturbed in the presence of a substrate based on the results of pocket RMSDs (Figure 7A and Table S2). Moreover, ligand binding results in a more spacious pocket compared to the corresponding apo one, which is consistent with the PCA results suggested by PC2.

The catalytic reaction of esterases belongs to the classic acid-base catalysis mechanism, which is mostly dominated by catalytic triads. As for EstJ6, residues (S146, E240, and H270) have been confirmed as catalytic triads [18]. In the catalytic process, an electron is transferred from E240 to S146 via H270 through the hydrogen-bond network among them. Then, H270 accepts H^+^ from Ser146, which generates an alkyl oxide ion whose nucleophilicity is much stronger than that of the original hydroxyl group to attack the carbonyl carbon of DBP. Therefore, the orientation of triads is very important for the catalytic reaction. Herein, the side-chain distances for S146-H270 and E240-H270 pairs were calculated during the last 100-ns trajectories of complex systems (Figure 7C,D). Interestingly, it was obtained that the average S146-H270 distances were 5.34 ± 0.39 Å for the WT-com system, 4.96 ± 0.38 Å for Mut-com, and the average E240-H270 distances were 6.87 ± 1.05 Å for the WT-com, 6.3 ± 1.01 Å and for the Mut-com, respectively. Combining the distance means and distribution plots delineates that compared with the wild-type system, distal mutations result in the conformational shift of triads, which become closer to each other. Although this shift is minor, it might be enough to induce significant effects on catalytic efficiency [48].

### 3.5. The Discrepancy of Residue-Residue Communications among All Systems

In order to identify the conformational dynamics that influence distal mutations on EstJ6, community network analysis was performed using the Network-View [49] plugin in VMD [36]. This method estimates the emotional relevance among residues and classifies them into different sub-groups or communities. The inter-community message flow is determined as the time-average communication between communities during simulations, which unveils the strength of inter-community coupling, which is represented by the thickness of edges between the two communities.

**Figure 8:**
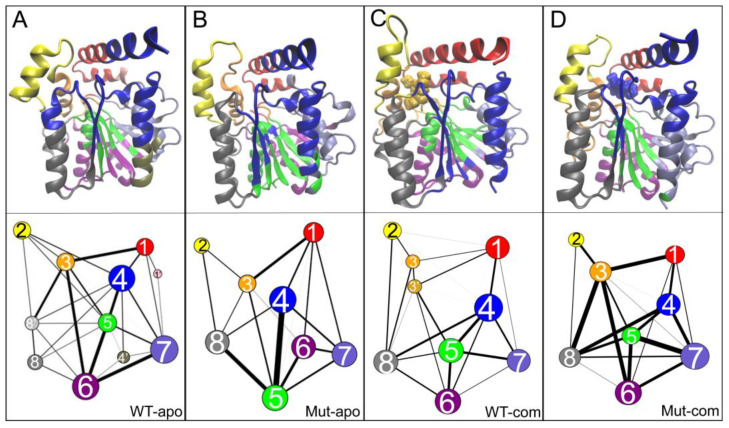
Community networks for all systems are displayed in the tertiary structure (top panels) or in a schematic two-dimensional representation (bottom panels) with corresponding colors: (**A**) WT-apo; (**B**) Mut-apo; (**C**) WT-com; (**D**) Mut-com. The size of a node is proportional to the number of residues within it, and the thickness of an edge is indicative of the communication strength of the two connected nodes. The number labels are given for communities of EstJ6 and ET2.2.

As illustrated in Figure 8, an interesting and highly reproducible community among all systems is group 2, which was mainly contributed by the lid region. Compared with the WT-apo system, it was found that the dynamic communications between group 2 with other regions decreased upon mutations. That is to say, the communication between the lid and the rest was weakened in Mut-apo, which might render more freedom for lid motion. This is consistent with our previous observations.

Generally, the number of communities can reflect intra-/inter-molecular coupling degrees during the dynamics process. The four systems vary in the number of communities. It is noted that an overall enhancement of intra-protein communication was observed for both mutant systems compared to the related WT one, as indicated by both the reduced number of communities and the reinforced betweenness among them. As for the two mutant systems, their protein dynamics networks share a very similar frame, but ligand binding highly strengthens the residue–residue coupling among communities. The enhanced communications around the ligand binding pocket might be favorable for substrate binding. Especially stronger communication between the substrate and the active center in the mutant system should increase the collision probability of reactants and further contribute to the improvement of enzyme activity.

Based on the community analysis, it could be observed that mutations allosterically affect the inter-communication of EstJ6. Combined with the differences discussed above, it explains the increased stability and hydrolysis capacity of the mutant in terms of dynamic coupling.

### 3.6. Distal Mutations Enhance the Binding of Substrate to EstJ6

After investigating the pocket stability in each system and triads and peculiarity in complex systems, we found that the distal mutations stabilize the active pocket of EstJ6. In view of the ligand features and the large pocket size of EstJ6, it is not easy to capture the transition state for catalytic reactions. Herein, we attempted to apply distance restraints to limit the sampling space of the substrate and triads.

**Figure 9:**
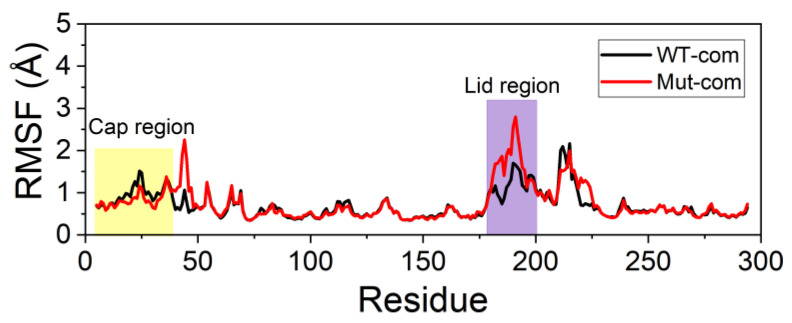
Cα RMSFs based on the last 100-ns distance-limited MD trajectories.

First, the convergence of MD trajectories was monitored by Cα RMSDs referring to their initial coordinates (Figure S6). Second, to explore the effects of mutations on the flexibility of the protein in detail, we calculated the RMSFs based on the last 100-ns MD trajectories (Figure 9 and Figure S7). It can be noticed that the lid region in the mutant still exhibited higher flexibility than the WT one, which is consistent with no-restraint simulations. The high flexibility of the lid improves the exchange efficiency of the product and reactant. Most residues around the binding pocket are rendered slightly in blue, which means that the pocket is a little more rigid in Mut-com: especially the cap region protecting the hydrophobic pocket. In order to harvest dominant conformations, cluster analysis was performed over the last 100-ns trajectories. Both systems were divided into two major clusters, accounting for 66% and 33%, respectively. Based on the representative structure of the most dominant cluster, the carbonyl carbon of DBP in the mutant system was more vulnerable to attack by S146 (Figure S8).

To further explore the allosteric effects of distal mutations on the binding of DBP to the receptor, the MM-GBSA method [34] was used to measure the binding free energy for DBP-bound systems. The average binding free energy, as well as the contributions of various energy terms, are shown in Table 1. As can be seen, compared with the wild-type system, DBP binding was enhanced upon mutations. In detail, the critical and powerful contributions to the binding free energy mainly came from the van der Waals and electrostatic interactions, and the nonpolar solvation interaction also played a slightly beneficial role. Then, the representative structures of the complex systems were used for molecular docking by the Autodock vina [26]. The mutant system presents a lower docking score than the WT one, which is consistent with the MM-GBSA results (Table 1). Therefore, it is certified that distal mutations make significant allosteric consequences on the binding of DBP.

**Figure 10:**
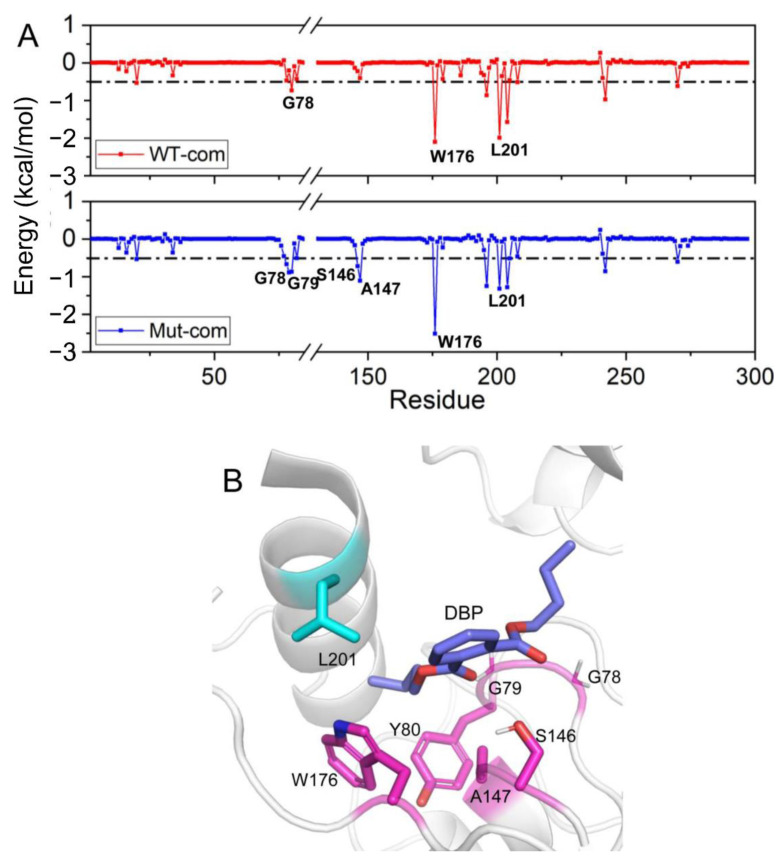
Residue contribution to the binding of DBP: WT-com and Mut-com (**A**), and the key residues are marked. (**B**) The key residues contributing to DBP binding are displayed in sticks.

As we all know, the per-residue contribution has been widely applied to investigate the details of protein-ligand interactions at the atomic level, and the computational results demonstrate good relevance with the experimental binding free energy differences upon alanine mutation. Here, by decomposing the total binding-free energy to individual residues, we attempted to further recognize hotspot residues (per residue contribution > 0.5 kcal/mol) that were responsible for ligand binding (Figure 10 and Table S3). For Mut-com, six residues (G78, G79, Y80, S146, A147, and W176) experienced larger contributions than those in the WT-com system. The increased contributions of these residues mainly originated from hydrophobic interactions. L201 close to the lid region plays a larger role in DBP binding in the WT-com system. However, because of the high flexibility of the lid, the binding with L201 is not conducive to the stability of the substrate.

### 3.7. Rational Designed Disulfide Bonds Increase the Thermostability of EstJ6

Based on the above analysis, we have learned how distal mutations upgrade the enzymatic properties of EstJ6, while thermostability is another essential factor for the application of industrial enzymes. Disulfide bonds are vital for the folding, stability, and function of many proteins, and the introduction of disulfide bonds is a common strategy in the rational design of proteins or enzymes to improve thermostability [50]. Hence, here we intend to adopt disulfide bond engineering to elevate EstJ6 thermostability.

Potential residue pairs that may form disulfide bonds were predicted using a web-accessible method DbD2 [37] (http://cptweb.cpt.wayne.edu/DbD2/index.php, accessed on 29 December 2021). There are 35 residue pairs that satisfy the default criteria of DbD2 (Table S4). Predicted substitutions may be located in essential functional areas that possibly harm catalytic processes. Hence, residues within 10 Å of the catalytic triad and 5 Å of the other important regions (such as the lid region and binding pocket) were excluded. After filtering, 16 potential residue pairs were prepared for reasonability assessment. The P217 and A224 pair was located downstream of the lid region and hence might influence the lid motion. The alteration of residues A250 and L262 may not be conducive to the correct positioning of H270. High conservative residues, including A53, A58, P167, and P231, were abandoned to minimize detrimental effects on enzyme activity.

**Figure 11:**
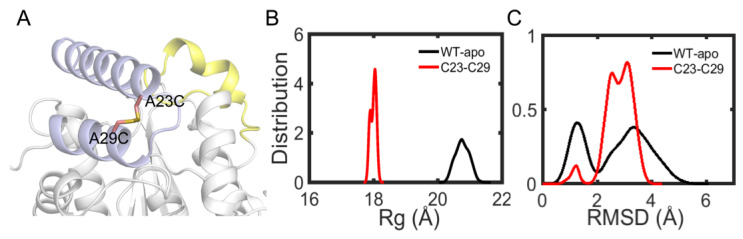
Structure and dynamics information of mutants. (**A**) The mutation sites A23C and A29C are depicted in sticks. (**B**) The distribution plot of *R*_g_ for the overall structure including wild type and mutant^A23C-A29C^. (**C**) The RMSD distribution plot of the cap region for wild type and mutant^A23C-A29C^.

Among the remaining 11 potential residue pairs, based on sequence and structure information, the A23C-A29C disulfide bond was chosen as a promising candidate for site-directed mutagenesis to improve the thermostability of EstJ6. Notably, this mutated pair is located at the cap region, which is a cover over the binding pocket (Figure 11A). The cap region accounts for pocket hydrophobicity and substrate selectivity [51]. Sequence alignment result suggests that the sites mentioned above often are hydrophilic residues. These residue substitutions may cause a negligible impact on substrate identification.

Finally, an A23C/A29C mutant was modeled with the introduction of a disulfide bond between them, and molecular dynamics simulations were applied to investigate protein stability. First, we analyzed the *R*_g_ of the overall structure. As Figure 11B shows, a more compact structure emerged after the introduction of the disulfide bond. Then, in order to further investigate the stability of the cap region, the RMSDs of this region were calculated. As depicted in Figure 11C, the new disulfide bond improves the stability of the cap region. These performances are similar to ET2.2 in their stability aspect. In the future, these mutations need to be further validated by biochemical and biophysical experimental characterization.

## 4. Discussion

Mutations, including distal ones, are sophisticated for their conformational and functional regulation of proteins. Understanding the implied molecular mechanism is helpful for the further improvement of enzyme resources. In this work, we attempt to clarify how distal mutations improve the activity of an esterase via computational approaches. Based on the above observations, the two following aspects deserve to be discussed further.

### 4.1. The Conformational Changes of the Lid Closely Related to the Activity of Esterases

The lid region, which is by most lipases and some esterases, controls the opening and closing of the substrate binding pocket. The outward motion of the lid makes the pocket accessible, allowing substrates to enter the active site and be hydrolyzed [52]. Researchers found that the movement tendency of the lid region influenced the substrate binding channel in lipase [53]. Hence, much research has focused on modifying the lid to improve the properties of the lipase. For example, Karkhane [54] and Panizza [55] applied mutations on the lid region of lipase and harvested mutants with enhanced activity. Li et al. developed an activity-enhanced esterase mutant with an unstable lid-like structure [56].

In this study, we also observed that the lid region in the EstJ6 mutant showed higher dynamics and a stronger tendency to move away from the active site, further proving that the lid region is a key factor affecting esterase activity. In the future, the rational design of esterases is suggested to focus on lid reconstruction for activity improvement and substrate specificity.

### 4.2. The Potential and Challenge of Computation Biology in Rational Design of Proteins

Natural enzymes often need to be improved or optimized for large-scale industrial applications. In order to overcome the problems of existing enzymes with poor features and the difficulties in mining new ones, researchers generally modify and optimize the existing ones via protein evolution. Right now, rational design employing computational approaches provides an incisive modification scheme with time- and labor-saving screening features. With the development of protein engineering methodologies and in silico tools, the discovery of new enzymes has resulted in increased industrial interest.

In the past, active sites were the major focus of enzyme design. The application of local modification strategies, focusing on chemical steps through transition state stabilization in the active sites of existing enzymes, played a pleasing role in the improvement of stability, activity, and substrate selectivity [57,58]. In addition, the platform Rosetta [59], invented by the David Baker group, started a new road for protein rational design. Based on this platform, many de novo-designed enzymes came into existence [60,61].

Mutations, located all around the enzyme structure, are also very common in many laboratory-evolved enzymes. Increasing evidence indicates that distal mutations are also significant for protein function, such as increased activity and drug resistance [62,63,64]. As indicated by many recent studies, the conformational dynamics of enzymes are crucial for substrate binding, product release, as well as allosteric regulation. Hence, unlike active-site mutations directly influencing ligand binding or activity, distal mutations usually produce marked effects on the redistribution of protein conformational states or the inner-protein residue interaction network and then alter the enzyme function. Dramatic changes in protein conformational stabilities after mutation, including distal ones, favor conformational states that are vital for the preferred functionality.

Of note is that mutations located all around the enzyme structure contrast with most of the computational design strategies that reduce the problem into active-site alterations [21]. Hence, it is promising to comprehend the effect caused by distal mutations on protein engineering, as reported by Shaikh et al. [65]. Now considering the rapid growth of computer performance and the gradual recognition of the molecular mechanisms involved in enzyme design, it is likely that rational design, including distal-site modifications, will eventually make a very significant contribution to protein engineering.

## 5. Conclusions

In the present study, we investigated the possible allosteric regulation mechanism of distal mutations on EstJ6. Our results demonstrate that distal mutations play a critical role in the dynamical behavior of EstJ6, including the opening motion of the lid region, as well as the strengthened residue coupling around the pocket. The opening of the pocket entrance should benefit the entering of the substrate and the exit of the product. Additionally, stronger ligand binding, accompanied by the enhancement of local hydrogen-bond networks involved in catalytic reactions upon mutation, might greatly increase the occurrence of catalytic reactions. Hence, our present study fills the gap between distal mutations and improved enzyme activity. As rational design has become the most rapid evolution method for the development of industrial enzymes, next, we in silico designed a new EstJ6 mutant with the introduction of a disulfide bond based on sequence and structure information to improve protein stability. Fortunately, further simulations confirm our prediction. These findings are of instructive significance for the exploration of new highly efficient industry esterases.

## Figures and Tables

**Table 1:** Terms of the binding free energy between WT-com and Mut-com averaged over the last 20 ns of the replicate runs (kcal/mol).

Contributions	WT-com (kcal/mol)	Mut-com (kcal/mol)
ΔE_vdw_	−41.34 ± 4.24	−44.50 ± 0.55
ΔE_ele_	−7.37 ± 6.98	−12.07 ± 10.13
ΔG_pol,sol_	19.85 ± 6.35	23.02 ± 5.83
ΔG_npol,sol_	−5.83 ± 0.43	−6.24 ± 0.09
ΔE_MM_	−48.71 ± 9.14	−56.57 ± 10.19
ΔG_sol_	14.02 ± 6.26	16.78 ± 5.92
ΔG_total_	−34.68 ± 4.47	−39.79 ± 4.70
Docking score	−5.5 ± 2.12	−6.45 ± 0.92

## Data Availability

Not applicable.

## Supplementary Materials

The following supporting information can be downloaded at: https://www.mdpi.com/article/10.3390/biom13030443/s1. Figures S1 and S2 and Table S1: Sequence conservative analysis for EstJ6; Figure S3: The Ramachandran map of EstJ6 structure predicted by Swiss-model server; Figure S4: The hydropathicity scores of EstJ6 residues; Figure S5: Root-mean-square deviations (RMSDs) of protein Cα atoms in the four systems; Figure S6: Cα Root-mean-square deviations (RMSDs) in simulations with distance restraints; Figure S7: Changes in RMSF upon mutations for complex systems during distance restricted simulations; Figure S8: The first representative binding state of complex systems; Table S2: The means and deviations of RMSD and *R*_g_ values of pocket residues; Table S3: The binding free energy contribution of essential residues in the pocket; Table S4: Predictions of potential disulfide bonds in EstJ6; Supplementary Video S1.mp4: Detailed motions along PC1; Supplementary Video S2.mp4: Detailed motions along PC2.
