# Supplementary material for: Computational Insights into the Allosteric Modulation of a Phthalate-Degrading Hydrolase by Distal Mutations
Source: Biomolecules. 2023 Feb 26;13(3):443. doi: 10.3390/biom13030443 (PMC10046322; doi:10.3390/biom13030443)
Supplement: Supplementary file 1 [file biomolecules-13-00443-s001.zip › biomolecules-2093296-supplementary.pdf]

Supplementary information

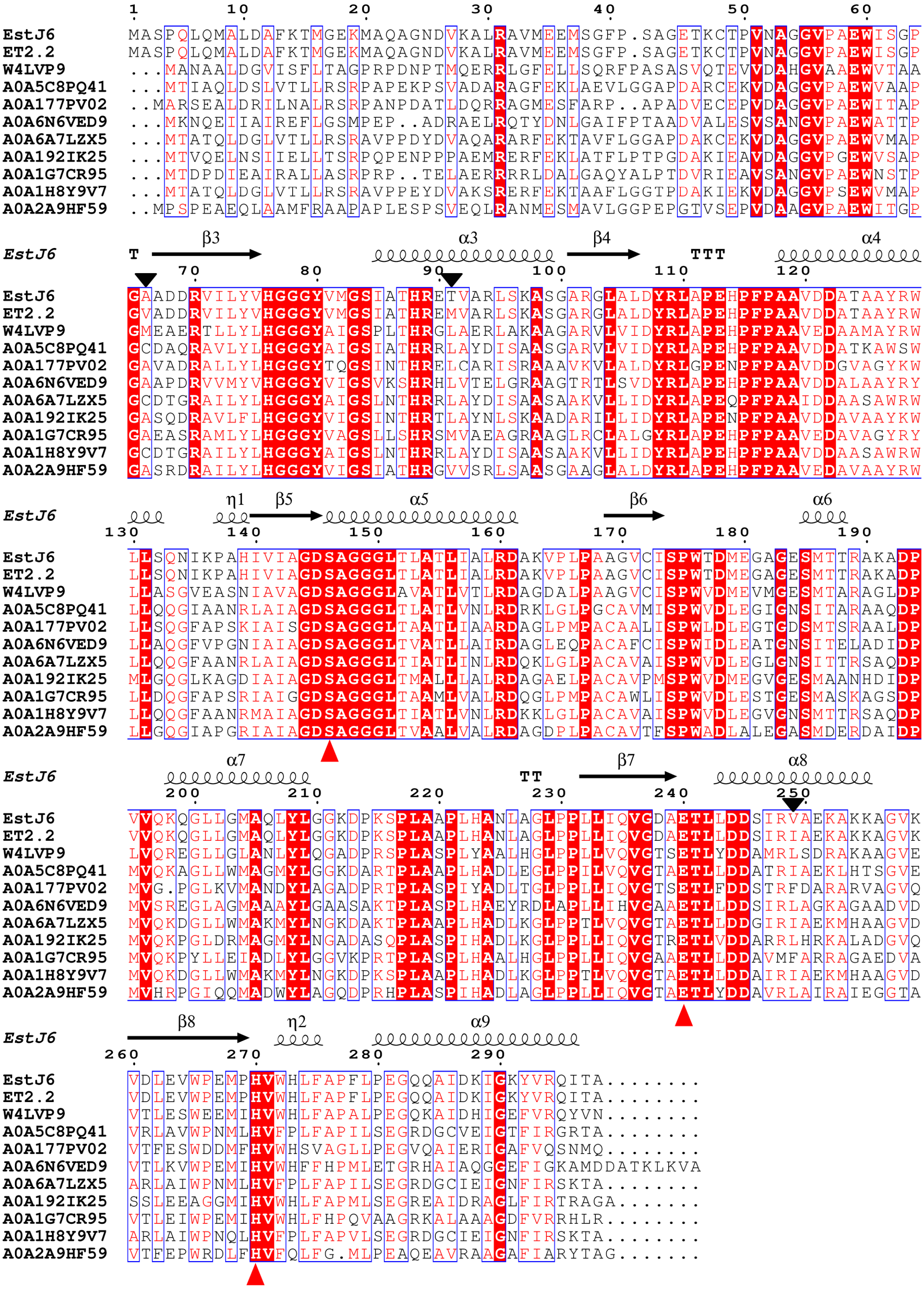

Figure S1. Sequence alignment of EstJ6 and its homologous proteins from the family IV (sequences similarity ≥ 57%). Highly conservative residues are shading in red. The mutated sites and triads are indicated by black and red triangles, respectively.

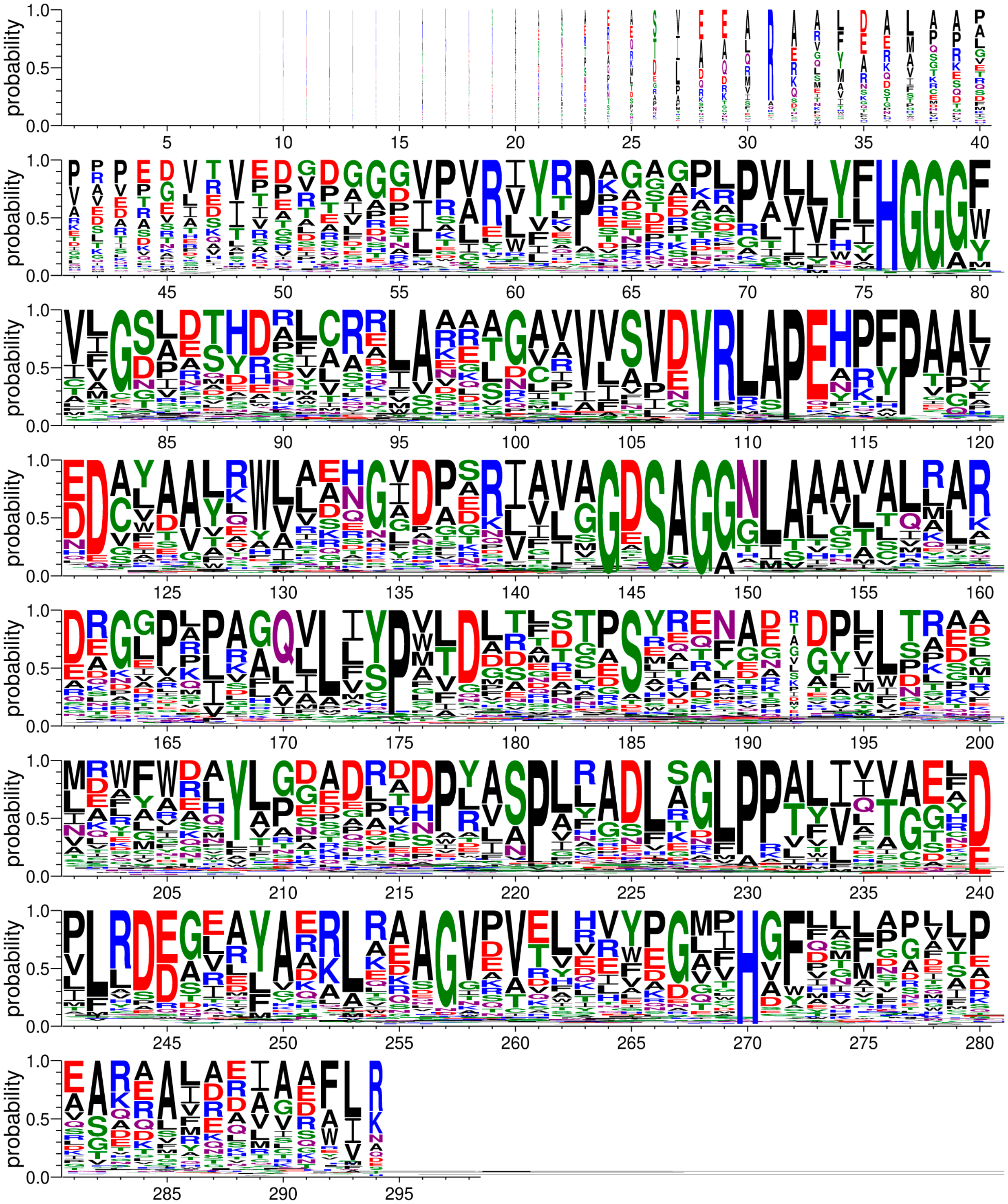

Figure S2. Sequence logo presentation of full-length EstJ6.

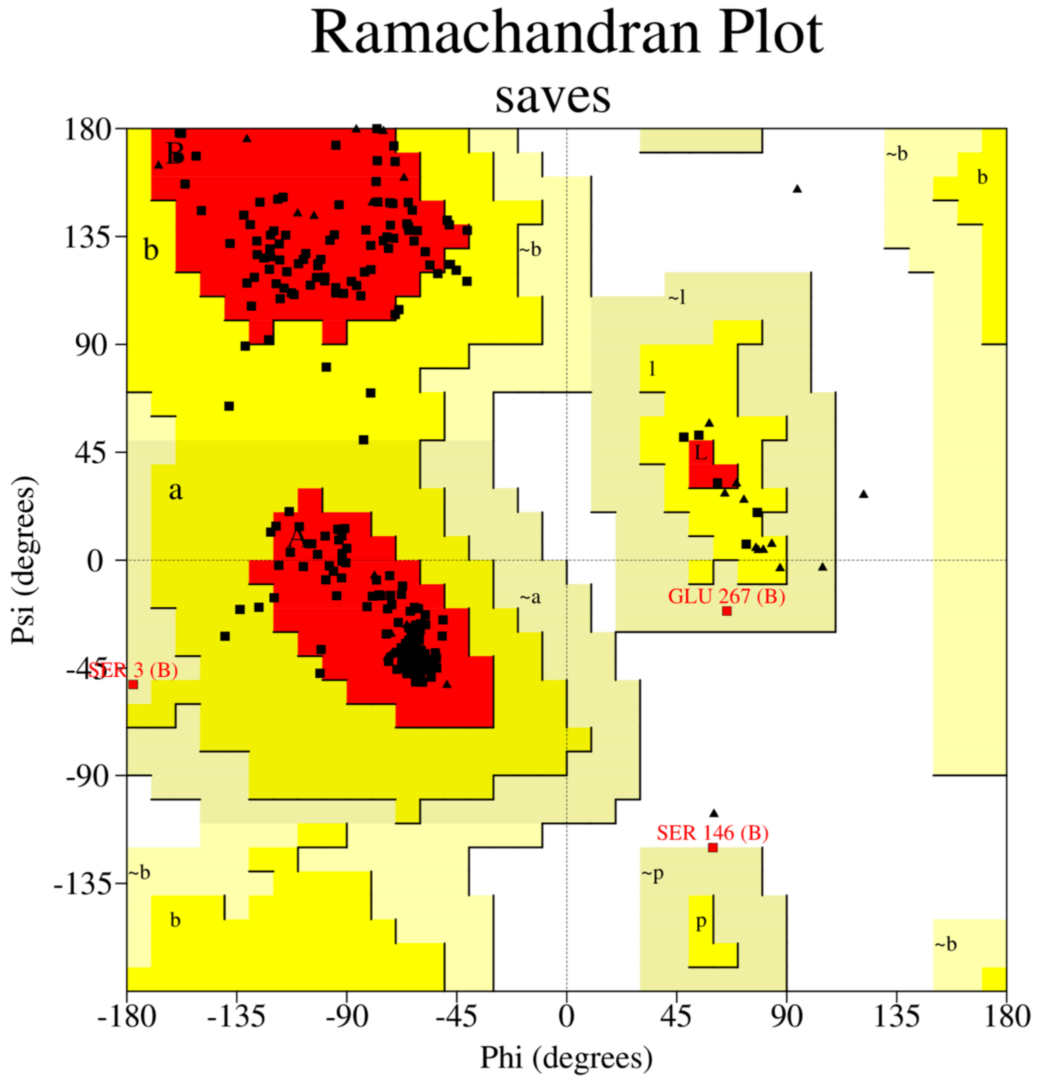

Plot statistics

Residues in most favoured regions [A,B,L]	220	90.9%
Residues in additional allowed regions [a,b,l,p]	19	7.9%
Residues in generously allowed regions [~a,~b,~l,~p]	3	1.2%
Residues in disallowed regions	0	0.0%
	----	------
Number of non-glycine and non-proline residues	242	100.0%
Number of end-residues (excl. Gly and Pro)	2	
Number of glycine residues (shown as triangles)	30	
Number of proline residues	22	

Total number of residues	296	

Figure S3. The Ramachandran map of EstJ6 structure predicted by Swiss-model server. Statistical data shows 90.9% residues reside in the most favored regions, which indicates the rationality of model structure.

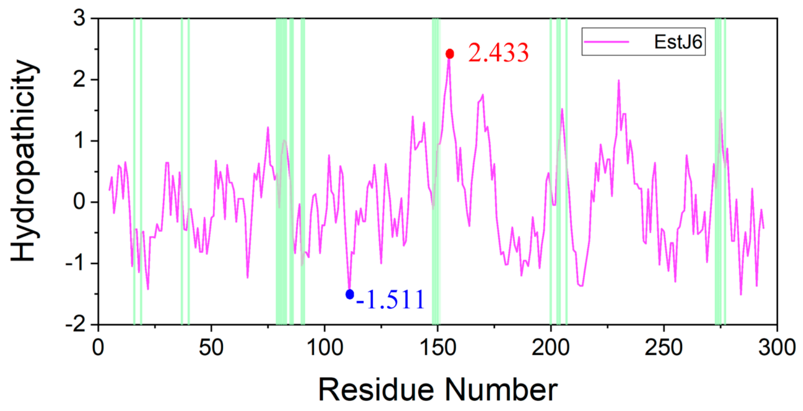

Figure S4. The hydropathicity scores of residues in EstJ6. The min- and max-values are -1.551 and 2.433, respectively. The green shadow indicates pocket residues.

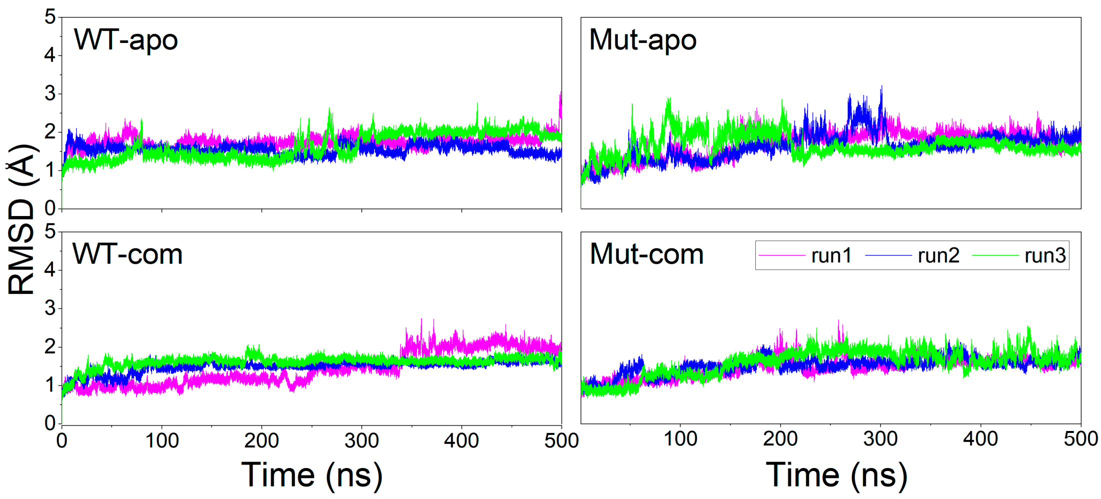

Figure S5. Root-mean-square deviations (RMSDs) of protein Cα atoms in the four systems.

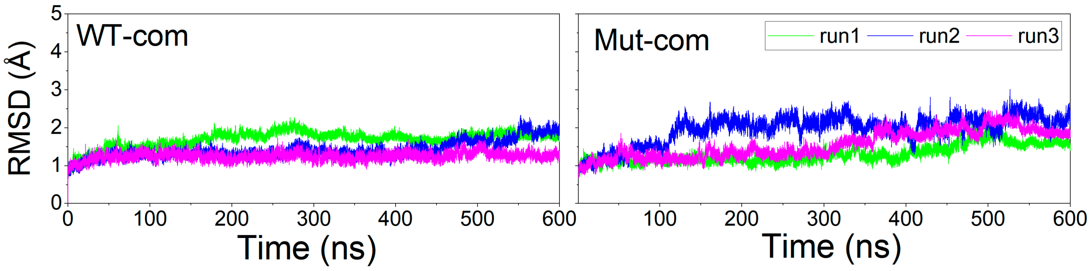

Figure S6. Cα Root-mean-square deviations (RMSDs) in simulations with distance restraints.

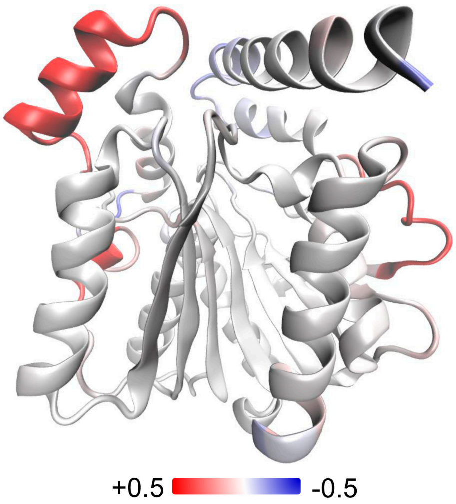

Figure S7. RMSF differences upon mutations for complex systems with distance restraints. The differences were mapped onto protein structure, displayed according to a color scale (lower and higher flexibilities depicted in blue and red, respectively).

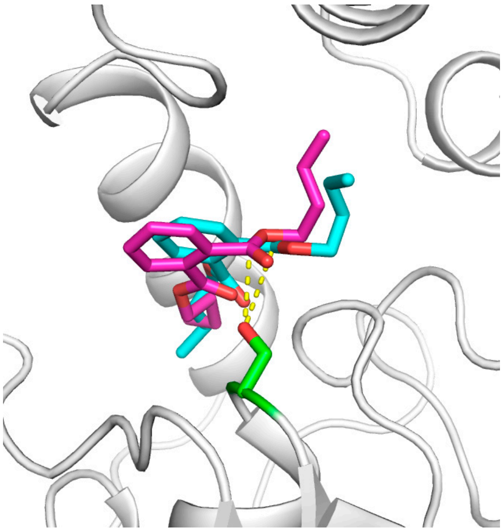

Figure S8. The dominant states of DBP^{WT} (cyan) and DBP^{Mut} (magenta) in the active site. S146 is shown as green sticks. The distance from the carbonyl carbon of DBP to the side-chain oxygen atom of S146 is 3.8 Å in WT-com and 3.6 Å in Mut-com

Table S1. The frequency of several crucial residues and mutation sites of ET2.2 (0%: least conservative, 100%: immutability).

Residue		Frequency
**Catalytic triads**	**S146**	97.17%
	**E240/D240**	23.88%/74.13%
	**H270**	97.18%
**Other crucial conservative residues**	**H76**	94.73%
	**G77**	95.2%
	**G78**	94.53%
	**A147**	82.75%
**Mutations**	**A67**	11.23%
	**T91**	4.53%
	**V249**	2.1%

Table S2. The means and deviations of RMSD and R_g values of pocket residues.

Systems	Pocket-RMSD (Å)	Pocket-R_g (Å)
**WT-apo**	2.12 ± 0.16	9.33 ± 0.37
**Mut-apo**	1.71 ± 0.17	9.26 ± 0.16
**WT-com**	2.09 ± 0.30	9.58 ± 0.18
**Mut-com**	2.08 ± 0.23	9.56 ± 0.20

Table S3. The binding free energy contribution of essential residues in the pocket.

	WT-com (kcal/mol)	Mut-com (kcal/mol)
**G78**	-0.47 ± 0.53	-0.67 ± 1.08
**G79**	-0.21 ± 0.30	-0.89 ± 0.40
**Y80**	-0.74 ± 0.16	-0.87 ± 0.23
**S146**	-0.23 ± 0.17	-0.72 ± 0.49
**A147**	-0.41 ± 0.09	-1.11 ± 0.68
**W176**	-2.11 ± 0.33	-2.52 ± 0.70
**L201**	-1.99 ± 0.63	-1.32 ± 0.18

Table S4. Prediction of potential disulfide bonds in EstJ6. The residue pairs, within 10 Å of triads and 5 Å of lid region colored in red, are out of consideration for disulfide bond engineering. The blue ones represent high conservative residues, beyond 10 Å of triads and 5 Å of lid region.

Res1 ID	Res1 AA	Res2 ID	Res2 AA	Chi3	Energy	Sum B-Factors
13	PHE	194	PRO	-98.08	0.95	1.76
13	PHE	195	VAL	-104.8	6.02	1.75
23	ALA	29	ALA	79.71	4.37	1.76
26	ASP	29	ALA	95.41	1.96	1.75
53	ALA	58	ALA	86.07	0.35	1.87
54	GLY	125	ALA	-112.16	3.68	1.89
63	GLY	96	SER	-116.63	3.99	1.85
63	GLY	97	LYS	78.36	5.51	1.79
75	VAL	148	GLY	74.49	3.63	1.99
76	HIS	88	HIS	115.96	5.17	1.88
77	GLY	145	ASP	-90.95	1.79	1.96
80	TYR	208	TYR	95.14	2.81	1.88
99	SER	101	ALA	-106.55	3.19	1.89
108	TYR	122	ASP	105.89	2.88	1.92
109	ARG	114	HIS	-74.86	4.5	1.81
114	HIS	118	ALA	86.8	4.27	1.88
123	ALA	155	THR	125.85	2.01	1.96
140	ILE	167	PRO	-108.97	3.46	1.93
145	ASP	173	ILE	-89.99	2.39	1.95
149	GLY	172	CYS	95.2	5.2	1.99
153	LEU	221	PRO	-72.55	2.73	1.93
177	THR	222	LEU	108.17	7.38	1.9
179	MET	205	ALA	101.77	4.3	1.85
185	SER	243	LEU	85.28	4.79	1.86
192	ALA	239	ALA	-106.24	3.9	1.82
216	SER	219	ALA	116.97	2.96	1.88
217	PRO	224	ALA	102.73	2.23	1.87
226	LEU	256	ALA	-99.08	3	1.83
231	PRO	259	LYS	117.78	4.54	1.9
236	VAL	246	SER	-90.92	0.75	1.92
250	ALA	262	LEU	121.14	6.85	1.88
253	ALA	258	VAL	106.6	5.84	1.88
275	PHE	279	LEU	84.38	1.16	1.8
276	ALA	282	GLY	-78.65	0.28	1.86
279	LEU	282	GLY	87.48	2.61	1.84
